# Longitudinal pathways of associations between motor proficiency and physical fitness during earlier and later childhood: The NW-CHILD study

**DOI:** 10.1177/00368504241232515

**Published:** 2024-03-15

**Authors:** Anita Elizabeth Pienaar, Carli Gericke, Wilmarié du Plessis

**Affiliations:** 1 Physical Activity, Sport and Recreation (PhASRec), Faculty of Health Sciences, Potchefstroom Campus, 56405 North-West University, Potchefstroom, South Africa

**Keywords:** Childhood, gender, fundamental movement, motor proficiency, physical fitness, physical education, public health

## Abstract

Understanding the relationships between motor proficiency (MP) and physical fitness (PF) is important for the future health of children, although longitudinal findings of this nature are limited. This study explored the association between MP and PF during earlier childhood (6 and 9 years old) and later childhood (12 years old) in boys and girls. A stratified and randomized research design including a baseline and two follow-up time-point measures (2010, 2013, and 2016) were used. Primary school children (N = 374, boys = 178; girls = 196) were tested with the Bruininks-Oseretsky Test of Motor-Proficiency-2, Short Form, and the Test of Gross Motor Development- 2, at ages 6 and 9 years, and with the Progressive Aerobic Cardiovascular Endurance Run test at age 12. Spearman Rank Order Correlations and stepwise regression analyses were used to analyze the data. Although of a low magnitude, proof of a dynamic longitudinal, but a stable relationship between MP and PF were found and with different gender-specific relationships in this pathway of association. A small but significant percentage of variation in PF at 12 years could be explained by overall motor competence (MC) and object control skills (OCS) at the ages of 6 and 9 years. Among girls, the association with PF at 12 years was influenced by both OCS, and MC, although only MC contributed to the variation found in boys. Socio-economic status made an insignificant contribution at 6 years to later PF in boys, but not in girls. MC, including OCS during early and middle childhood can be considered as possible triggers of physical activity which again, can increase PF during later childhood. Obtaining early competence in these developmental areas is therefore important to promote positive and sustainable trajectories of health with long-term health outcomes.

## Introduction

A lack of motor competence (MC), low physical fitness (PF), and physical activity (PA) are associated with various undesirable health outcomes such as childhood overweight and obesity.^1,2^ During later childhood, good MC and PF are furthermore considered key enablers to successfully generalize different capabilities in sport-specific and specialized skill environments.^2^ Evidence that links MC and PF to positive cognitive functioning and academic outcomes and the subsequent future wellbeing of children is also appearing more frequently.^3–6^ Other benefits that are positively associated with health-related PF include physical health; psychological wellbeing, and cognitive vitality^2,7–9^ PF is therefore a physiological state of wellbeing that not only reduces the risk of hypokinetic disease; but also provides a foundation for participation in sports that again contributes to an active lifestyle.

MC is a global term that describes the degree of skilled performance, movement control, and coordination in a wide range of motor skills, including fundamental and specialized movements that underly a motor outcome. These movements are essential for tasks like sports, physical activities, and daily life.^10–13^ Outcomes of MC are influenced by movement quality, control, and coordination.^12,13^ Health-related fitness is comprised of cardiorespiratory endurance, muscular strength and endurance, body composition, and flexibility which is usually related to disease prevention and health promotion.^14–16^ In the context of MC, coordination and physical qualities are closely linked as physical attributes like strength, flexibility, balance, and endurance serve as the building blocks upon which coordination skills are shaped and refined.^12,13^ Coordination is directly influenced by adequate strength such as in jumping as strength forms the foundation for muscle stability and control and thereby allows individuals to perform coordinated movements effectively. Good balance again ensures that the body remains steady and controlled, especially in activities that involve complex or dynamic movements, such as during evasive movements or during catching and kicking skills in game-like situations,^12^ or even when performing everyday activities like climbing stairs. When continued efforts are required such as in long-distance running, it is necessary to maintain coordinated movements over an extended period without fatigue-induced loss of control. Aerobic fitness and MC are therefore considered complementary in an individual's ability to engage in physical activities, improve overall health, and excel in sports or recreational pursuits.^13^ It can be assumed that, even though MC and PF are theoretically distinct constructs, they are closely linked.^13^ The level of physical qualities of MC can therefore either facilitate or hinder an individual's ability to perform coordinated movements effectively while also influencing fitness levels.^17^

Studies report positive associations between MC and PA in children.^18–20^ It is suggested that developing a certain level of MC would facilitate successful participation in a variety of physical activities, specifically vigorous activities, which again provide subsequent opportunities for health-related fitness development.^14,20^ Children with low MC often display low levels of PA, resulting in lower PF levels.^21,22^ Lacking MC can therefore serve as a constraint of PA behavior as it can influence the desire to be physically active or to be physically fit and thereby provide a potential barrier for the progression of PF trajectories. Preliminary evidence is provided of an MC proficiency barrier that can impact PF based on findings of low MC relative to what was found in older adults.^20,23^

If children therefore fail to acquire adequate levels of MC during the earlier childhood years, participation in physical activities, enjoyment of sports activities, and health-related PF might become compromised later in life.^21–24^ After studying the influences of several factors, healthy PF was the only significant contributor to elementary school student's participation in physical activities.^25^

The association between MC and PF in children, however, still needs more understanding. Studies do report positive associations between cardiorespiratory fitness, muscle strength, and endurance.^16,26^ Researchers^10,17,27^ also report that abilities within MC are significant predictors of PF. An Australian study,^15^ found in this regard that object control skills (OCS) at 10 years were associated with adolescent cardiorespiratory fitness at 16 years, accounting for 25.9% of the variation in fitness. This association can be influenced by gender, as a higher association was reported in girls (28%) compared to boys (18%).^28^ Gender differences were also reported where the locomotor component of MC revealed the strongest effect on PF in Brazilian girls, 7 to 14 years old, while locomotor and OCS had larger effects in younger boys and stability in older boys.^29^ Components of MC therefore proved to be significant predictors of PF with locomotor skills being the strongest predictor.^29^ It is, however, reported that this relationship might change across childhood as the correlation between OCS skills and health-related fitness is found to increase over time with a positive relationship between MC and PF reported across childhood and early adolescence.^29^ A systematic review, however, concluded that associations between MC and health-related PF, are, in general, the same in boys and girls.^26^

Apart from age and gender influences, socio-economic status (SES) is also found to be associated with the relationship between MC and PF. Researchers confirmed the influence of socio-economic inequalities and unique environmental influences in different countries on PF.^30,31^

The benefits of MC to PF are strong from cross-sectional studies demonstrating the protective effects of MC on unhealthy PF^27,29,32,33^ and on long-term health outcomes in children.^15,17,18,27,30,32,34^ The predominantly cross-sectional nature of the available evidence can, however, not confirm causal pathways between MC and PF. Limited studies are also available to confirm longitudinal associations in young children as similar associations that were studied focussed primarily on the middle childhood to adolescent period.^15,18,28,29^ Researchers also report that the dynamic relationship between MC and measures of PF across age has not yet been investigated comprehensively and urge more longitudinal research in this regard.^13,16,29,32–35^ These gaps in our understanding of how this dynamic association between MC and PF changes across age in typically developing children from early childhood through middle to later childhood, motivated this study. This study therefore investigated pathways of association between MC and PF during earlier, middle childhood (ages 6 to 9 years) and later childhood (age 12) in South African (SA) children of both sexes residing from different SES backgrounds.

## Methods

### Study design

The Health Research Ethical Committee of the Faculty of Health Science at the North-West University granted permission for the North-West Child Health, Integrated with Learning and Development longitudinal study (NW-CHILD Study) (NWU-00070-09-A1). The research was completed following The Code of Ethics of the World Medical Association (Declaration of Helsinki) for experiments involving humans. The reporting of the methodology of the study is according to the STROBE guidelines.

A longitudinal research design was utilized, and data was collected over seven school years (2010–2016) in the North West Province of South Africa (SA). This provided follow-up data of 6 years, including one baseline and two time-point measurements, in 2010 (grade 1: 6 years old), 2013 (grade 4: 9 years old) and 2016 (grade 7: 12 years old). The research group was selected employing a stratified randomized study design in conjunction with the Statistical Consultation Services of the North-West University, Potchefstroom, SA. To recruit participants, a list of all the schools in the North West Province was obtained from the Department of Basic Education in the North West Province. Stratification was done for school districts, school quintiles (1–5), and gender (male and female). All the schools in this province are grouped into eight educational districts, each consisting of 12 to 22 regions with approximately 20 schools (minimum 12, maximum 47) per region. Twenty schools from four different educational districts, representing five different school quintiles, and children of both sexes, were recruited to take part in the study. A poverty classification is used by the North West Department of Basic Education to classify schools in different quintiles (quintile 1, i.e., school types from low SES backgrounds, to quintile 5, i.e., school types associated with higher SES backgrounds). The classification system of the National Census information was used, which included income, ratio of dependents ratios, and literacy levels.^36^ Quintiles 1 and 2 schools are the poorest schools and are exempted from paying any school fees. The North West Province of SA is characterized by high poverty, especially in rural areas, unequal distribution of income between different population groups, and unemployment. All typical developing children with no physical or mental disabilities or health issues that could prevent them from participating in the PACER test were eligible for inclusion in the sample. Written parental or legal guardian consent and child assent were obtained before testing. All children who met these inclusion criteria, whose parents or legal guardian granted written parental consent, had to provide verbal (when younger than 8 years), or written assent, based on their age before they were allowed to participate in the study. The testing procedures were conducted at schools during the mornings of a school week as arranged and confirmed by the headmaster of each school.

#### Participants

In 2010, 880 children in their grade 1 year, were recruited for the baseline measurements of the study. Eight hundred and sixteen children, 419 boys and 397 girls completed the baseline measurements. All of these children were invited to again participate in the follow-up measurements in grade 4, and in grade 7.

The number of participants with complete datasets at baseline (2010) and the two follow-up time-point measures (2013 and 2016) included 374 participants (N* *= 374). This group comprised 178 boys (47.59%) and 196 (52.41%) girls. Two hundred and fourteen of these participants (90 boys (50.57%) and 124 girls (63.27%)) were in quintile 1–3 schools representing low SES settings, while 160 participants (88 boys, 49,44% and 72 girls, 36.73%) represented higher SES school settings (quintile 4 and 5). The mean age of the group was 6.84 years in grade 1, 9.9 ± 0.42 years in grade 4, and 12.9 ± 0.41 years in grade 7.

The loss to follow-up of subjects over the study period was 442 (54.1%). Migration from schools, lack of written parental consent, or absence on the day of testing were the main reasons for such a large loss of participants over the 7-year school period. All children with physical disabilities, without parental consent or who have not been part of all three time point measurements were excluded from the study.

### Measuring instruments

#### Bruininks-Oseretsky Test of Motor-Proficiency, Second Edition Short Form (BOT-2SF)

The BOT-2SF^33,37^ was used to determine the participant's MC during the baseline and the follow-up time-point measurements (2010, 2013, and 2016). This norm-based instrument is suitable for use in 4- to 21-year-olds.^37^ The BOT-2SF evaluates motor skills in four area components and consists of the following: fine motor skills (divided into fine motor precision and fine motor integration); hand coordination (divided into hand agility and upper limb coordination); body coordination (divided into bilateral coordination and balance); and the strength and agility component (divided into running speed, agility, and strength). The BOT-2SF, which is validated against the full version consists of 14 items, was used to evaluate the MC skills. Raw scores were converted to point scores and gender-specific standard scores (SSs) and converted to age-equivalent descriptive categories and percentile values. The *BOT-2* is a reliable test battery with values that vary between *r *= 0.7 and *r *= 0.8 for different age groups, with sufficient validity to provide effective measurements of gross and fine motor skills.^37^ Excellent psychometric properties (α = 0.92) and strong test-rest reliability are also reported for the BOT-2.^38,39^ The BOT can be considered as a composite score that is built on a wide range of test items from different motor abilities or motor domains and was therefore used to assess MC from a more broadly defined perspective as it includes locomotor, nonlocomotor, and functional coordination activities.

Data was collected by trained postgraduate students and senior researchers in Kinderkinetics. All test items were individually administered, and one tester was assigned to score all the participants in a single test item throughout to eliminate influences of inter-rater variability. Trained interpreters explained the test instructions to the participants whenever the mother tongue of the participant was neither English nor Afrikaans.

#### Test of Gross Motor Development, Second Edition (TGMD-2)

The TGMD-2 is a valid, norm-referenced measure designed to test gross motor functioning of children 3 to 10 years old.^40^ The test consists of 12 motor skills, divided into two subtests, namely locomotor (run, hop, gallop, leap, horizontal jump, and slide) and OCS (overhand throwing, catching, underhand rolling, dribbling, kicking, and striking a stationary ball).^40^ Only the OCS subtest was used for the purpose of this study to determine proficiency in six OCS of children aged 6 and 9 in grades 1 (2010) and 4 (2013). The OCS skills subset that is part of the TGMD-2 obtains a more comprehensive representation of the different OCS that are used in SA sports and more specifically in the school sports system of this country as the BOT-2 SF. All the test items of the TGMD were set up in a way to be assessed in a specific order with the tester sitting in one place. One tester, demonstrator, and interpreter were assigned to the OCS station and all the participants were then scored in all the OCS by this one tester to eliminate influences of inter-rater variability. A trained interpreter was also used to explain the test instructions to participants if they did not understand the instructions. After a visual demonstration by the demonstrator of the skills, two attempts were allowed, and the scores were added together. Each skill consists of several behavioral components that represent a mature pattern of the skill. A score of 1 is awarded for a correct performance and a 0 for an incorrect performance. SSs for each of the subitems are calculated from the raw scores and then converted to gender and age-specific percentiles and a SS for OCS.^40^ The descriptive competence ratings for subtest SSs in the TGMD-2 manual are indicated as very superior (17–20), superior (15–16), above average (13–14), average (8–12), below average (6–7), poor (4–5), and very poor (1–3). The TGMD-2 has a validity of *r* = 0.89.^40^ This test battery was not used during the 2016 measurements as it is only validated for children up to 10 years.

#### Progressive Aerobic Cardiovascular Endurance Run test (PACER)

Participants completed the Progressive Aerobic Cardiovascular Endurance Run (PACER); a multistage progressive cardiorespiratory endurance test that is part of the FITNESSGRAM testing protocol.^41^ Participants ran back and forth between two lines spaced 20m apart while having to keep pace with a pre-recorded audio cadence. The test begins with an easy pace and increases in difficulty as the time between the audio beeps becomes shorter. When a participant failed to reach the line in time with the cue on two consecutive attempts, or voluntarily dropped out due to fatigue, the test was terminated. The number of laps completed, was then recorded, and then converted to an age estimated VO_2_ max according to age and gender. More details on the procedure can be found in previous literature.^36^ This test was only used during the time-point measures at 9 and 12 years in 2013 and 2016 as it is not deemed to be a suitable test for younger children.^41^

### Statistical analysis

Statistical analyses were performed using Statistica for Windows 2019. Data was analyzed descriptively (percentages, frequency distributions, means, and standard deviations) to describe the group and MC and PF characteristics. A sample size calculation utilizing Statistica showed that N = 148 is sufficient for an R-square of 0.1, and N = 250 for an R-square of 0.06, for 4 predictors with 90% power and an alpha = 0.05. The Spearman Rank Order Correlation was used to determine the association between MC and PF during early (6 years), middle (9 years), and later (12 years) childhood and to determine associations with gender and SES. This analysis was selected as it is a nonparametric correlation not assuming normality. The effect size cut-offs as set by Cohen^42^ were used to determine practical significance. A *r *≈* *0.1 is interpreted as a small effect, *r *≈* *0.3 as a medium/moderate effect, and *r *≥* *0.5 as a large effect size. Stepwise regression analysis was used to determine the strength of the association between MC and OCS mastery and PF during earlier and later childhood including gender and SES. The percentage variance explained is indicated by the following cut points, R^2^ = 1% is interpreted as a small effect, R^2^ = 10% as a medium effect, while R^2^ ≥ 25% is considered as a large effect. For statistical significance, p is set at ≤ 0.05.

## Results

Table 1 displays the descriptive statistics of the BOT-2 and the TGMD-2 that were used in grade 1, at age 6, and in grade 4, at age 9, to determine the general MC and OCS proficiency of the group and also by gender. The PF of the group obtained by the PACER at ages 9 and 12 years are also displayed. Boys compared, to girls, displayed higher mean BOT standard total scores in grades 1 (44.43 vs 39.25) and 4 (51.46 vs 48.56). The standardized, gender OCS SS increased in boys and girls from age 6 (7.15 vs 7.43) to age 9 (8.67 vs 9.62). Boys also displayed a higher number of PACER levels at ages 9 (25.46 vs 17.91) and 12 years (37.76 vs 21.78) and in the gender-specific estimated VO_2_ max of the PACER when compared to girls (43.53 vs 40.88, age 9; 44.45 vs 38.90, age 12).

**Table 1.** Descriptive characteristics by group and per gender of the BOT-2 standard score (ages 6 and 9), the TGMD-2 (ages 6 and 9) and the PACER and VO_2_ max (ages 9 and 12).

Grade	Gender/group	*M*	*SD*	Min	Max
BOT-2 SF standard score (grades 1 and 4)					
Age 6	Boys	44.43	5.99	32.00	63.00
	Girls	39.25	6.06	26.00	63.00
	Group	41.72	6.55	26.00	63.00
Age 9	Boys	51.46	7.25	35.00	75.00
	Girls	48.56	8.01	34.00	73.00
	Group	49.94	7.78	34.00	75.00
TGMD-2 (grades 1 and 4)					
Age 6	Boys	7.15	2.32	2	13
	Girls	7.43	2.30	2	14
	Group	7.30	2.31	2	14
Age 9	Boys	8.67	2.24	2	13
	Girls	9.62	2.41	4	15
	Group	9.16	2.38	2	15
PACER					
Age 6	N/A—Not assessed in grade 1				
Age 9	Boys	25.46	13.85	4.00	74.00
	Girls	17.91	8.79	2.00	59.00
	Group	21.56	12.11	2.00	74.00
Age 12	Boys	37.76	17.57	7.00	83.00
	Girls	21.78	12.83	5.00	80.00
	Group	29.40	17.22	5.00	83.00
VO_2_ Max					
Age 6	N/A—Not assessed in grade 1				
Age 9	Boys	43.53	4.92	35.70	60.30
	Girls	40.88	3.03	35.70	54.60
	Group	42.15	4.25	35.70	60.30
Age 12	Boys	44.45	6.17	33.10	60.30
	Girls	38.90	4.62	32.60	59.10
	Group	41.54	6.07	32.60	60.30

Note. BOT-2: Bruininks-Oseretsky Test of Motor-Proficiency, Second Edition; M: mean; Max: maximum; Min: minimum; PACER: Progressive Aerobic Cardiovascular Endurance Run; SD: standard deviation; TGMD-2: Test of Gross Motor Development, Second Edition.

Table 2 displays the results of a correlation analysis that determined the relationship between MC and the PACER levels in the group at ages 9 and 12 years old, and also per gender. Statistically significant correlations were found between the BOT total at 12 years and PF scores at both ages. A small, but significant relationship was found between MC and PF at the age of 9 (*r* = 0.26), and this relationship increased to moderately significant (*r* = 0.35) at the age of 12 years. Girls showed a small but significant relationship between MC and PF at ages 9 (*r* = 0.16) and 12 (*r* = 0.31), while this relationship was strong and moderate in boys at both ages (*r* = 0.31) and (*r *= 0.38).

**Table 2.** Correlations between MC (BOT-2SF standard score) and PF (PACER) at ages 9 and age 12 by group and by gender.

		9 years	12 years
Measure	Year	PACER (*r*)	PACER (*r*)
BOT-2SF SS 2016 (12 years)	Boys	0.31*	0.38*
	Girls	0.16*	0.31*
	Group	0.26*	0.35*

Note. r ≈ 0.1 small effect, r ≈ 0.3 medium effect, r ≥ 0.5 large effect. OCS not assessed at 12 years. BOT-2SF: Bruininks-Oseretsky Test of Motor-Proficiency, Second Edition Short Form; MP: motor proficiency; OCS: object control skills; PACER: Progressive Aerobic Cardiovascular Endurance Run; PF: physical fitness; SS: standard score.

The contribution of MC at age 6 and 9 years to PF at age 12 years was analyzed through a stepwise regression analysis (Table 3).

**Table 3.** A stepwise regression analysis of explained variances in PF at age 12 by MC, OCS, gender, and SES at ages 6 and 9 years.

*N *= 374	Steps entered	b*	Standard error of b*	b	Standard error of b	p-value	R^2^-change
Age 6 on age 12 PACER (R^2^^ ^= 0.271)							
Intercept				−3.510	5.116	0.493	
Gender	1	0.395*	0.049*	13.604*	1.715*	0.001*	0.215*
BOT-2SF SS	2	0.189*	0.053*	0.497*	0.139*	0.001*	0.047*
OCS SS	3	0.104*	0.490*	0.785*	0.370*	0.035*	0.009*
Age 9 on age 12 PACER (R^2^^ ^= 0.248)							
Intercept				−0.138	5.513	0.980	
Gender	1	0.451*	0.048*	15.513*	1.636*	0.001*	0.214*
BOT-2SF SS	2	0.150*	0.047*	0.332*	0.105*	0.002*	0.028*
OCS SS	3	0.084	0.047	0.607	0.345	0.079	0.006

Note. R^2^-change—coefficient of determination; b*—value of the slope; p-value—significance; p ≤ 0.05. BOT-2SF: Bruininks-Oseretsky Test of Motor-Proficiency, Second Edition Short Form; MC: motor competence; OCS: object control skills; PACER: Progressive Aerobic Cardiovascular Endurance Run; PF: physical fitness; SES: socio-economic status; SS: standard score.

PF was not measured at age 6; therefore, this analysis was only performed on the data obtained at ages 9 and 12 years. The results showed that 27.1% of the variance that was found in the PF at age 12 can be explained by the results at age 6, where gender makes a significant contribution of 21.5% to the variance. The BOT-total contributes to an additional 4.7% (p < 0.05) and the OCS SS add an additional 0.9% (p < 0.05). At age 9, 24.8% of the variance in PF at age 12 years can be ascribed to gender (21.4%), MC (2.8%) and OCS (0.6%), although the contribution of OCS was not significant at this age. The strength of the association between MC and PF at ages 6 and 9 years, with older ages during later childhood, including gender in the stepwise regression analysis, showed a large significant effect (R^2^ ≥ 25%).

As gender revealed a main influence on the overall explained variance at ages 6 and 9 years, (21.5% and 21.4%), separate stepwise regression analyses were conducted for boys and girls (Tables 4 and 5).

**Table 4.** Stepwise regression analysis of explained variance by MC at ages 6, 9, and 12 of PF in girls.

*N *= 196	Steps entered	b*	Standard error of b*	b	Standard error of b	p-value	R^2^-change
Age 6 girls on age 9 girls PF (PACER) *(R^2^^ ^= 0.042)*							
Intercept				12.161*	2.107*	0.000*	
OCS SS	1	0.204*	0.071*	0.772*	0.270*	0.005*	0.042*
Age 6 girls on age 12 girls PF (PACER) *(R^2^^ ^= 0.086)*							
Intercept				−0.979	5.871	0.868	
BOT-2SF SS	1	0.192*	0.074*	0.404*	0.156*	0.010*	0.062*
OCS SS	2	0.164*	0.074*	0.935*	0.420*	0.027*	0.024*
Age 9 girls on age 12 girls PF PACER *(R^2^^ ^= 0.061)*							
Intercept				0.845	6.049	0.889	
BOT-2SF SS	1	0.204*	0.072*	0.326*	0.115*	0.005*	0.051*
OCS SS	2	0.101	0.072	0.539	0.386	0.164	0.009

Note. R^2^-change—coefficient of determination; b*—value of the slope; p-value—statistical significance; p ≤ 0.05. BOT-2SF: Bruininks-Oseretsky Test of Motor-Proficiency, Second Edition Short Form; MC: motor competence; OCS: object control skills; PACER: Progressive Aerobic Cardiovascular Endurance Run; PF: physical fitness; SS: standard score.

**Table 5.** Stepwise regression analysis of the explained variance by MC at ages 6, 9, and 12 years of PF in boys.

*N *= 178	Steps entered	b*	Standard error of b*	b	Standard error of b	p-value	R^2^-change
Age 6 boys on age 9 boys PF (PACER) *(R^2^^ ^= 0.041)*							
Intercept				2.976	8.530	0.728	
BOT-2SF SS	1	0.201*	0.078*	0.464*	0.181*	0.011*	0.025*
SES	2	0.133	0.078	3.700	2.165	0.092	0.016
Age 6 boys on age 12 boys PF (PACER) *(R^2^^ ^= 0.062)*							
Intercept				5.438	9.603	0.572	
BOT-2SF SS	1	0.249*	0.073*	0.728*	0.214*	0.001*	0.062*
Age 9 boys on age 12 boys PF (PACER) *(R^2^^ ^= 0.033)*							
Intercept				14.183	9.880	0.153	
BOT-2SF SS	1	0.141	0.076	0.342	0.185	0.066	0.026*
OCS SS	2	0.088	0.076	0.691	0.599	0.250	0.007

Note. R^2^-change—coefficient of determination; b*—value of the slope; p-value—statistical significance; p ≤ 0.05. BOT-2SF: Bruininks-Oseretsky Test of Motor-Proficiency, Second Edition Short Form; MC: motor competence; OCS: object control skills; PACER: Progressive Aerobic Cardiovascular Endurance Run; PF: physical fitness; SES: socio-economic status; SS: standard score.

These analyses confirmed associations between early MC and PF in both genders. In girls (Table 4), OCS at 6 years significantly explained 4.2% of the variance that was found at age 9 in PF while MC and SES showed no association. Both MC (step 1) and OCS (step 2 at 6 years), however, entered as significant steps in the regression analysis to explain PF at 12 years. The contribution of these variables to PF at 12 years showed that 8.6% of the overall variance in PF can be significantly ascribed to general MC (6.2%) and OCS (2.4%) while SES did not enter in the regression analysis. The contribution of the same variables at age 9 to the variation that was found in PF at age 12 was slightly lower (6.2%) but again included MC (5.1%, p < 0.05) and OCS (0.1%, p < 0.05). SES did not contribute to the explained variance in girls. MC entered higher during later childhood while OCS entered during early and later childhood. It can be seen that good OCS probably contributes to girls’ higher MC at 9 years because both MC and OCS contributed to the PF of 12-year-old girls. Table 5 reports the same analyses for boys.

Surprisingly, OCS did not enter at 6 years compared to in girls where it explained 4.2% of the variance at 6 years. In boys, 2.5% of the variance was explained by MC at 6 years. SES also contributed to the explained variance in boys (p > 0.05), although only at a young age and for a short time period (only until age 9 years).

The contribution of MC at age 6 in boys and girls was the same at 12 years (6.2%). However, MC and OCS both influenced the PF of girls which was not the case in boys. The contribution of MC and OCS to PF in girls was greater at 12 years (8.6% vs 6.2%). MC also made similar high and significant contributions to the explained variance at ages 6 and 9 to explain PF at age 12 in boys (2.5% and 2.6%, respectively). The percentage variance explained in boys and girls were both more than R^2^ = 1% which can be interpreted as a small significant effect.

## Discussion

This study investigated pathways of associations between overall MC and OCS in earlier childhood at ages 6 and 9 years with PF during later childhood at age 12. The findings confirmed that MC during earlier childhood is associated with PF during later childhood in both boys and girls although the magnitude of association was low. These findings are supported by both the correlation analysis and the stepwise regression that were performed, including gender as part of the stepwise regression analysis. The findings confirmed that gender contributed largely and consistently to the explained variation of PF from early childhood at age 6, and during middle childhood, at age 9 (21.5% and 21.4%, Table 3). The large contribution of gender motivated a separate stepwise regression analysis for boys and girls. The contribution of general MC as assessed by the BOT-2 total, OCS as assessed by the TGMD-2, and SES, were analyzed in the stepwise regression analyses. These analyses (Tables 4 and 5) showed that MC during earlier and middle childhood had a small (R^2^^ ^> 1.0), although a significant and similar effect on PF during later childhood in both boys and girls with a contribution in both of 6.2%. The OCS of girls at 6 years, however, made an additional 2.4% contribution to the explained PF association during later childhood at 12 years, where an overall explained variance of 8.6% was found in girls, which was not the case among boys. In boys, OCS did show a small association up to age 9 years, as it entered the stepwise regression during middle childhood at age 9, although only on a nonsignificant level (0.07%).

Very few longitudinal studies were done in this field on same-aged children to compare our findings with. It is, however, consistent with the findings on Portuguese children^18^ where children with better gross motor coordination at age 6, based on the total score on the four KTK-test items, had higher fitness and activity levels at 10 years compared to those displaying poorer motor coordination. Researchers^15,28^ also reported similar, although higher associations between MC (more specifically OCS), and PF at 10 and 16 years of age. The association between MC and PF is, also consistent with cross-sectional findings^29^ showing that the relationship between overall MC (more specifically studied as locomotor and OCS) and health-related PF is strong and stable across childhood and early adolescence in both boys and girls. This association is also confirmed through a recent systematic review showing strong evidence of a positive association between MC, cardiorespiratory fitness, and musculoskeletal fitness in children and adolescents.^26^ A longitudinal study^22^ comparing children with low MC with typical developing children also confirmed that individuals with low MC exhibit inferior overall PF levels compared to peers with higher MC. Other studies that were also of cross-sectional nature, reported that cardiorespiratory fitness, power, muscle strength, and endurance are all positively associated with MC.^21,26^ In addition, other researchers^17,27^ concluded that MC components prove to be significant predictors of PF and that PA levels can explain the association between MC and PF.

The contribution of each of the studied variables was found to be different in boys and girls over the shorter time from early to middle childhood. In girls, OCS was the only step that entered early childhood at age 6 to play a role in PF during middle childhood (age 9) (4.2%). This finding illustrates the overarching and subsequently important role of OCS, especially in girls, to trigger PA and thereby put girls early in their lives on an active trajectory that is conducive to their fitness. It is suggestive that competence in such skills combined with the development of a positive perception of competence in sports aid girls at younger ages to play ball skill-related games which might again contribute to vigorous play and activity and subsequently PF.^43^ In SA, school sport includes netball that requires catching and throwing skills which is played by girls from an early age. Participation in mini-netball, an age-adapted form of netball is already introduced to girls in grade 1. As a result, young girls can become part of netball teams, as participation in team sports is found to be conducive to PF outcomes.^43^ This early influence of OCS on girls remained, although the influence weakened, while the influence of general MC increased over a longer period. Over the follow-up period from 6 to 12 years, the role of general MC strengthened and entered as a first step showing a 6.2% contribution to PF, while OCS made a 2.4% additional contribution to the explained variance. Longitudinal findings on associations between MC and PF during childhood and adolescence in Australian children,^15^ also confirmed that OCS at 10 years is associated with adolescent cardiorespiratory fitness in both boys and girls, accounting for as much as 25.9% in the variation of fitness at 16 years. A higher association was also found in girls (28%) compared to in boys (18%) in an earlier Australian study,^28^ which agrees with our findings of a higher association of OCS in girls. Cross-sectional findings on 546 Brazilian children, aged 7 to 14 years showed that locomotor motor skills trigger their involvement in PA while boys need locomotor, OCS, and MC for involvement in motor activities.^29^ In our longitudinal study, this conclusion might be more relevant to the findings of girls as OCS showed a different but also higher and significant association, not only between early and middle childhood (4.2%), but also between earlier and later PF (2.4%) than in boys. The results of Luz et al.,^29^ however, confirmed the findings of our study regarding the relationship between MC and PF. The surprisingly poorer association between early OCS and later PF in boys compared to girls can possibly be ascribed to a generally higher interest among all boys in doing movement skills associated with OCS and applying these skills during play which might contribute to more vigorous physical activity and subsequently improved PF in most boys. Differences are reported in this regard in the way that boys and girls grow up and are supported to participate in sports, resulting in differences in the frequency of training and exposure to instructions for a specific skill. Researchers^43,44^ also reported differences between 1 and 9-year-old children where boys showed better OCS and girls better locomotor skills.^43,44^ Although speculative, based on the above reasoning, but also based on the results of researchers^35^ that showed that children with low MC are not as physically active as their typical peers, there might be a bigger differentiation between girls who are object control proficient compared to those with poorer OCS competence. This can exclude girls with poorer OCS from involvement in early team sport, leading to less exposure to environments where such skills can improve and subsequently lesser vigorous PA that is conducive to PF. In agreement, it is reported that a lack of experience in developing various motor skills is undeniably linked to a lack of participation in various moderate-to-vigorous physical activities, including structured and unstructured play and sports that again can promote cardiovascular fitness.^45^ In general, studies reported declining PA levels in girls from 10 years of age which is not conducive to their PF and subsequently their health.^1^ A study also found that health-related PF was the only significant contributor to elementary school students’ participation in PA from several factors tested.^25^ Active participation and increased PA among boys and girls are also associated with sport participation although boys are reported to be more likely to belong to sports teams than girls.^43,44^ This may therefore impact their PF levels positively as an enabling factor for PA and vice versa thereby providing the physical foundations to enjoy engaging in a variety of physical activities.^23^ Our findings therefore highlighted that it is especially important to equip all girls with optimal OCS at a young age as this motor skills repertoire can again motivate them to participate in sport and active play which again can trigger PA, and subsequently PF.

SES only showed an association with the PF of boys up to middle childhood, as it entered the stepwise regression as a second step, although on a nonsignificant level (0.016%, Table 5). Other researchers also confirmed the influence of socio-economic inequalities and unique environmental influences in different countries on physical activities.^30,31,44^ Large practical significant differences were also reported between the OCS proficiency of boys from low (poorer) and high SES school environments^43^ although these findings are based on 9-year-old children.

Our findings, therefore, reveal different pathways of associations with cardiovascular fitness in boys and girls from an early age. OCS at age 6 showed significant associations with PF at age 9 in girls and not in boys, while the contribution of MC along with OCS in combination also strengthened from ages 6 to 12 in girls. In boys, only MC, and not OCS, was significantly associated with PF at the ages of 9 and 12 years. This relationship strengthened from early childhood in both genders which agrees with other studies that also report strengthening effects of the relationship,^13,29^ although MC was the main contributor rather than OCS. The MC that was assessed in this study included items assessing overall coordination, hand-eye-coordination, balance, strength, running speed and agility and even fine motor skills, suggesting that a wider range of motor proficiencies contribute at older ages to partaking in PA that is beneficial to PF levels, and especially in boys. In agreement, researchers report that failure to acquire adequate levels of competence in a broad foundation of motor skills during childhood and adolescence restricts avenues for participating in health-enhancing physical activities, thereby presenting a potential barrier to subsequent progression of cardiovascular fitness and MC trajectories.^32^ As locomotor skills were not assessed in the TGMD-2 but were part of the BOT-2 test items that were assessed, it might suggest that the contribution of overall and functional coordination and different components of locomotor skills along with other components of health-related fitness, such as strength, are important to contribute to the relationship with PF at older ages, and therefore needs further attention from researchers. These findings therefore contest the hypothesis that OCS in comparison to locomotor skills has a significant impact on health-related fitness, at least in boys which also needs further investigation.

It is anticipated that the knowledge gained by this study will bring to the fore the importance of enhancing both MC and PF in children as important building blocks for increased physical literacy, health, and wellbeing. The development of both MC and health-related PF at a young age may therefore promote positive and sustainable trajectories of health with long-term health outcomes. Both should also be considered important targets for early interventions to improve both physical activity and fitness in the early years. It should also be noted from a more specialized sporting environment perspective that physical attributes are highly specialized, signifying that not all individuals possess absolute MC in all aspects. Therefore, the development and maintenance of these physical attributes to improve MC should be considered through targeted training and conditioning programs.

The longitudinal nature of the study design is a strength of the study since it is one of few studies of this nature that enabled an in-depth look, spanning seven school years, into the association between MC, OCS, and PF during earlier and later childhood. MC was also assessed from a more broadly defined perspective using the BOT-2. The limitations of this study should, however, also be acknowledged. The study was bound to one of nine provinces of SA and to draw more comprehensive generalizations, similar studies should be extended to a more representative area. Although the study was based on a randomized research design, a power analysis was not performed, which is acknowledged as a limitation of the study. The loss of subjects over the study period was relatively large and we acknowledge that this limits the internal validity of the study findings. A sample size calculation however showed enough power to determine meaningful findings. A further limitation was that only the object skill subset of the TGMD-2 was used. If the locomotor subset was included, more direct comparisons with similar studies in this area was possible.^29^ Product-oriented measures such as the TGMD-2 may also be limited in their predictive utility**.** School quintile status was used as a proxy of SES in this study which also had certain limitations. It is recommended that the educational background and income of parents should rather be used as SES indicators. More similar and especially longitudinal studies are therefore recommended, based on our findings, to improve our understanding of other movement-related variances that can also explain PF in older primary school children, such as perceived MC or activity enhancement by means of involvement in an array of sports requiring different enabling abilities, which could not be explained by this study's findings.

## Conclusion

Overall, the findings are in concurrence with prior limited investigations, highlighting a longitudinal and dynamic interrelation between MC and PF. Although the magnitude of this association was low it exhibited constancy and manifested augmentation with advancing age, albeit following distinct gender-specific trajectories. The results suggest that early general MC in both genders, but especially OCS proficiency in girls, is key to consider as pathways of influencing positive activity behaviors. The period between 6 and 10 years emerges as a critical phase for the refinement of fundamental movement skills and the development of appropriate behaviors that may persist. Proficiency in fundamental motor skills can thus establish pivotal foundational prospects for skill refinement and transference, culminating in favorable health consequences for juvenile wellbeing. This improved comprehension of requisite general MC and OCS during the formative years underscores the necessity for forthcoming interventions that concurrently foster MC and health-related PF within developmentally appropriate contexts. Such an approach may be the most advantageous path to nurture both constructs and enhance and promote comprehensive functional capacities in children.
